# Observed and Expected Survival in Men and Women after Suffering a STEMI

**DOI:** 10.3390/jcm9041174

**Published:** 2020-04-19

**Authors:** Isaac Pascual, Daniel Hernandez-Vaquero, Marcel Almendarez, Rebeca Lorca, Alain Escalera, Rocío Díaz, Alberto Alperi, Manuel Carnero, Jacobo Silva, Cesar Morís, Pablo Avanzas

**Affiliations:** 1 Department of Cardiology, Central University Hospital of Asturias, 33011 Oviedo, Spain; ipascua@live.com (I.P.); marcel.almendarez@gmail.com (M.A.); lorcarebeca@hotmail.com (R.L.); alberto.alperi.garcia@hotmail.com (A.A.); cesarmoris@gmail.com (C.M.); avanzas@gmail.com (P.A.); 2 Research Institute of the Principado de Asturias, 33011 Oviedo, Spain; diazmendezro@gmail.com; 3 Department of Functional Biology, Physiology Area, Faculty of Medicine, University of Oviedo, 33006 Oviedo, Spain; 4 Cardiac Surgery Department, Central University Hospital of Asturias, 33011 Oviedo, Spain; alain_2623@hotmail.com (A.E.); jsilva8252@yahoo.es (J.S.); 5 Cardiac Surgery Department, San Carlos Clinic Hospital, 28040 Madrid, Spain; mcarnero@me.com; 6 Department of Surgery, Faculty of Medicine, University of Oviedo, 33006 Oviedo, Spain; 7 Department of Medicine, Faculty of Medicine, University of Oviedo, 33006 Oviedo, Spain

**Keywords:** myocardial infarction, life expectancy, gender

## Abstract

Introduction: Mortality caused by ST elevation myocardial infarction (STEMI) has declined because of greater use of primary percutaneous coronary intervention (PCI). It is unknown if patients >75 have similar survival as peers. We aim to know it stratifying by sex and assessing how the sex may impact the survival. Methods: We retrospectively selected all patients >75 who suffered a STEMI treated with primary PCI at our institution. We compared their survival with that of the reference population (general population matched by age, sex, and geographical region). A Cox-regression analysis controlling for clinical factors was performed to know if sex was a risk factor. Results: Total of 450 patients were studied. Survival at 1, 3, and 5 years of follow-up for patients who survived the first 30 days was 91.22% (CI95% 87.80–93.72), 79.71% (CI95% 74.58–83.92), and 68.02% (CI95% 60.66–74.3), whereas in the reference population it was 93.11%, 79.10%, and 65.01%, respectively. Sex was not a risk factor, Hazard Ratio = 1.02 (CI95% 0.67-1.53; *p* = 0.92). Conclusions: Life expectancy of patients suffering a STEMI is nowadays intimately linked to survival in the first 30 days. After one year, the risk of death for both men and women seems similar to that of the general population.

## 1. Introduction

Ischemic heart disease is the single most common cause of death worldwide and its prevalence is increasing, with variations between countries [1].

Short and long-term mortality caused by ST elevation myocardial infarction (STEMI) has significantly declined during past decades because of greater use of primary percutaneous coronary intervention (PCI), modern antithrombotic therapy, and secondary prevention measures [1,2]. Nevertheless, mortality remains high. In-hospital mortality of unselected patients with STEMI ranges from 4 to 12%. Mortality at one-year follow-up is approximately 10% [3,4]. Primary PCI has widely shown to be a life-saving procedure in the STEMI setting. However, several factors have an impact on mortality just after the STEMI. Age, sex, left ventricular function, or reperfusion therapy have been previously identified [1,2,3,4].

Age is an unmodifiable risk factor for mortality. It is known that patients >75 years old, even those treated with primary PCI, are at a higher risk of death. However, it is unknown whether this subgroup of patients may recover their life expectancy. Some studies have described long-term survival of patients with STEMI treated with primary PCI [3,5]. However, these results alone provide little information because the life expectancy of any population depends on the region of residence. In fact, there are significant differences in mortality even between industrialized countries. For instance, women 65 years of age have a life expectance 2 years longer in Spain than in the United States [6].

Moreover, sex is one of the key factors affecting the frequency and severity of acute myocardial infarction (MI). Several studies have shown a later onset of MI in women compared with men. Thus, MI occurs more often in men below 60 years and in women above 75 years [7,8,9,10]. A significant relationship between sex and age after acute MI has been demonstrated. On one hand, young women may have higher mortality than young men. On the other, mortality between both sexes seems similar in the elderly population [11,12]. In women, MI appears more frequently with atypical symptoms, which can delay primary PCI. In addition, the risk of bleeding during PCI and the presence of comorbidities in this group are higher and may have an impact on survival [13,14,15]. In this regard, previous works have shown that women with STEMI have higher short-term mortality than men [16,17].

However, there is an active debate regarding whether long-term survival could be worse in women. In many countries and geographical areas of the world, women have higher life expectancy than men. Therefore, comparing long-term survival curves in men and women after a STEMI would be insufficient [18,19,20,21]. The main objective of this study is to analyze whether patients >75 years of age with STEMI treated with primary PCI recover a life expectancy similar to that of the general population for the same age, sex, and geographical area, focusing on differences between sexes.

## 2. Material and Methods

### 2.1. Selection of the STEMI Sample and Construction of the Reference Population

We retrospectively selected all consecutive patients older than 75 years old suffering STEMI treated with primary PCI, from January 2014 to January 2020 at the Hospital Universitario Central of Asturias, in Spain. The primary PCI was performed for all patients who fulfilled the definition of STEMI following the ESC guidelines. This was defined as emergent PCI with balloon, stent or another approved device performed on the infarct-related artery without fibrinolytic treatment [1]. All procedures were performed in a catheterization laboratory by the same team of senior interventional cardiologists with more than 10 years of experience in primary PCI.

The reference population was constructed using mortality data of our Autonomous Region (Principality of Asturias) from the Spanish National Institute of Statistics (INE) stratified by age and sex. These data are available on the official INE website (http://www.ine.es/jaxiT3/Tabla.htm?t=27154). In order to be able to compare their late survival with that of the general population, all patients were matched by age and sex with the general population of the same geographical area. The whole process is described in the statistical section.

### 2.2. Data Collection

We retrospectively collected all data related to the baseline, intra-procedural, short-, and long-term follow-up periods from a digital database. One of the researchers collected all-cause mortality data during the follow-up by analyzing the information available in the medical records of the Hospital Universitario Central of Asturias.

Usually, patients were treated at discharge following the optimal medical treatment according to European Society of Cardiology Guidelines [1].

### 2.3. Primary Objectives

1. To compare the survival curves of patients with STEMI treated with primary PCI with that of the general population, matching by age, sex, and geographical region.
2. To compare the survival curves of patients who survive hospital discharge or first 30 days after the STEMI with that of the general population matching by age, sex, and geographical region.
3. To establish differences between men and women in the previous objectives.
4. To know if sex is a risk factor for long-term mortality.

### 2.4. Statistical Analysis

Quantitative and categorical variables were described as mean ± standard deviation (SD) and n (%), respectively. To compare survival of patients who suffered a STEMI with the general population of the same sex, age, and territory, we calculated the following estimations: (1) observed survival, (2) expected survival, and (3) relative survival (RS) [22,23,24]. The “strs” command of STATA^®^ v.15.1 (College Station, TX, USA) was used [25].

1. Observed survival is the real survival of the patients who suffered a STEMI and were treated by primary PCI at our institution. To estimate it, the usual Kaplan-Meier analysis was used.
2. Expected survival is considered the estimated life expectancy of a person from our region matched for the same characteristics (age and sex) of our STEMI patient. In other words, the theoretical survival of the patients if they had not suffered the STEMI. Its calculation was performed using the Ederer II method, which is the method of choice for the matching [26]. This method uses the mortality rates for different intervals of age, sex, and region provided by the INE [27]. If the expected survival is included in the 95% confidence interval of the observed survival, no differences were considered to exist.
3. Relative survival is an estimation of the survival that patients would have in the theoretical assumption that they could only die due to that STEMI or its consequences [25,28]. Its calculation is derived from the rate between the observed survival and the expected survival. For instance, a RS of 100% would indicate that the STEMI had no consequences on survival. Conversely, a RS of 80% during the first year would indicate that 20% (100–80%) of the patients who suffered the STEMI died because of this event or any of its consequences [22,23,24]. Therefore, if the confidence interval of the RS includes 100%, there is no evidence of mortality due to the STEMI and this would indicate that the primary PCI was completely effective in solving the problem [26,28].

In order to further study the influence of sex on late survival, a Cox regression analysis was performed. Sex was considered the independent variable and the following variables acted, from a theoretical point of view, as possible confounding factors: age, diabetes, hypertension, dyslipidemia, history of smoking, chronic renal dysfunction, previous acute myocardial infarction, previous coronary surgery, previous coronary angioplasty, lesion on anterior descending artery, multivessel disease, and Killip class 3 or 4. Hazard proportionality was evaluated by visual assessment of the ln (analysis of time) vs. -ln (-ln survival probability). The role of sex on late survival was evaluated taking into account the risk of being woman versus being a man (reference). A *p* value < 0,05 was considered statistically significant. All analyses were performed with STATA^®^ v.15.1 (STATA Corp, TX, USA). This study was conducted in accordance with the Declaration of Helsinki.

## 3. Results

### 3.1. Baseline Characteristics

During the study period, we included 450 consecutive patients with a STEMI, treated with primary PCI. Total of 438 patients out of 450 were Caucasian (97.3%); 263 (58.4%) patients were male. Female patients were slightly older (83.1 vs. 81.6 years old), with higher prevalence of hypertension and lower prevalence of smoking habit, previous MI and previous CABG. The rest of the baseline characteristics had no significant differences (Table 1).

### 3.2. Procedure and Discharge Data 

Radial approach was preferred with a similar distribution between men and women. The most frequent vessel affected was the left anterior descending artery in 218 patients (48.4%) followed by the right coronary artery in 136 patients (30.2%). Total of 308 (68.4%) patients were in Killip class I followed by 70 (15.6%) in class II (16.8%). Male patients had more multivessel disease 141 (53.6%) vs. 72 (38.5%) in women. Total of 38 (8.4%) of the patients had intra-procedural complications, 2 (0.4%) patients had vascular complications, 25 (5.6%) arrhythmic complications, 11 (2.4%) required mechanical ventilation, and 7 (1.6%) died during the procedure. At discharge, the mean LVEF was 49 ± 11% and 80 (17.8%) patients had a moderate or severe valvular heart disease (mitral or aortic). The peak ultra-sensitive troponin T was 5725 (±7410) pg/mL, 101 (24.2%) patients developed contrast induced acute kidney injury and there were 59 (13.1%) deaths during the admission or first 30 days. Detailed procedural and discharge data can be seen in Table 2 and Table 3.

### 3.3. Observed Survival, Expected Survival, and Relative Survival

The mean of the follow-up of the censored observations was 34.6 ± 21.4 months. Overall, there were 141 (31.3%) deaths, 86 (32.7%) were men and 55 (29.9%) were women. There were 31 (11.8%) and 28 (15.2%) deaths during the first 30 days after the STEMI in men and women respectively. All causes of death are described in Table 3.

**Figure 1:**
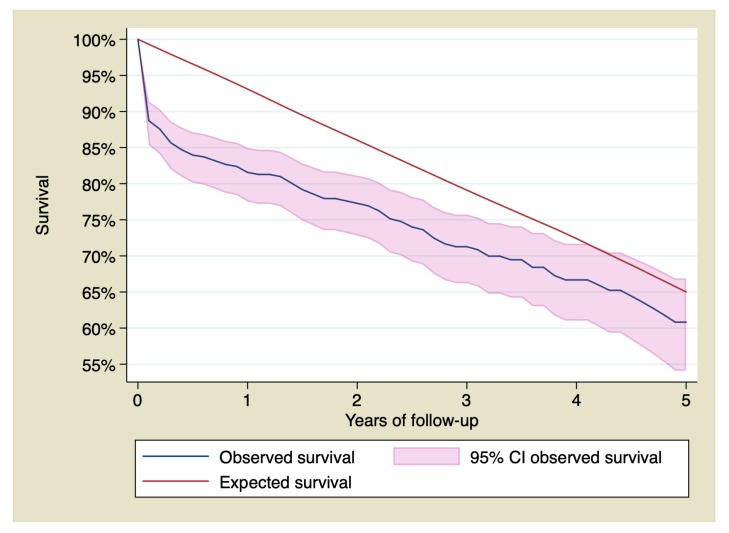
95% confidence interval of the observed survival compared with the expected survival of the sample. CI: Confidence Interval

Cumulative survival of patients with STEMI at 1, 3, and 5 years of follow-up was 81.56% (CI 95% 77.51–84.96), 71.27% (CI 95% 66.21–75.72), and 60.82% (CI 95% 54.09–66.88), being 93.11%, 79.10%, and 65.01% respectively for the reference population. Figure 1 shows survival of patients who suffered a STEMI treated with primary PCI compared with the reference group.

**Figure 2:**
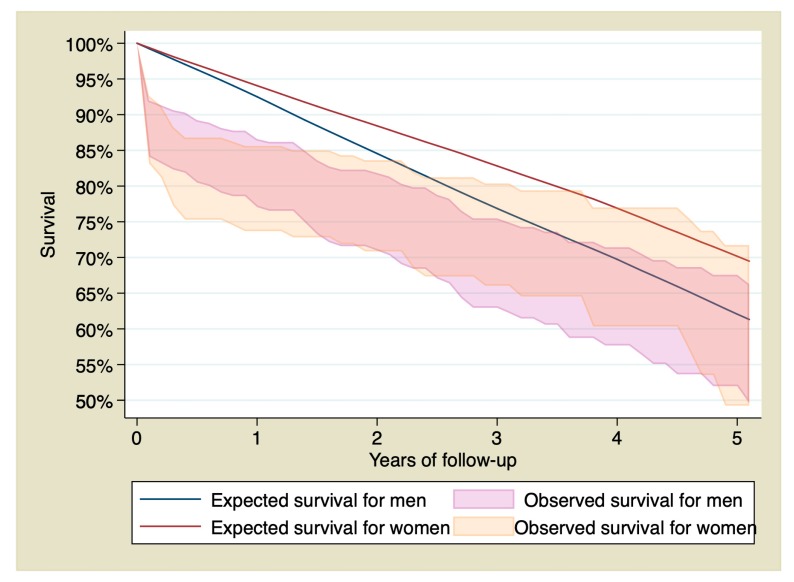
95% confidence interval of the observed survival compared with the expected survival of the sample stratified by sex.

Stratifying by sex, survival of men with STEMI at 1, 3, and 5 years of follow-up was 82.39% (CI 95% 77.05–86.59), 69.71% (CI 95% 62.95–75.48%), and 60.26% (CI 95% 51.97–67.58%), whereas in the reference group was 92.51%, 76.86%, and 62.06%. Survival of women with STEMI at 1, 3, and 5 years of follow-up was 80.44% (CI 95% 73.60–85.62%), 74% (CI 95% 66.04–80.37), and 61.57% (CI 95% 49.24–71.74), whereas in the reference group it was 94.06%, 82.81%, and 70.27%. Figure 2 shows observed and expected survival stratified by sex.

**Figure 3:**
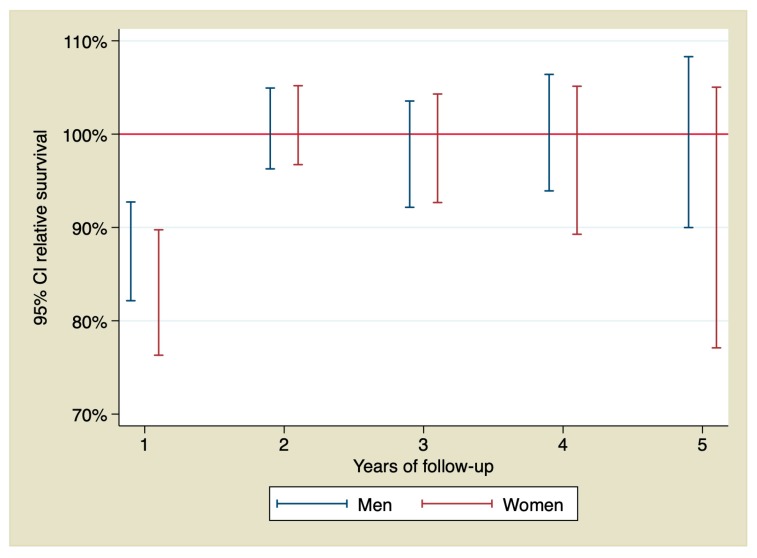
Relative survival by year of follow-up and stratified by sex.

RS in men calculated by annual intervals showed an excess of mortality due to the STEMI only during the first year of follow-up with a RS = 88.06% (IC 95% 82.15–92.73), indicating an excess of mortality of 11.94%. However, after that year, RS did not show an excess of mortality due to the STEMI. That means that observed and expected survival were similar. RS in women calculated by annual intervals showed an excess of mortality during the first year higher than the observed in men, with a RS = 83.89% (CI 95% 76.31–89.75) indicating an excess of mortality due to the STEMI of 16.11%. In the following years, there were no differences in excess of mortality due to the acute event either. RS by year of follow-up is shown in Figure 3. Table 4 shows observed and expected survival and RS stratified by sex and calculated by the year of follow-up.

### 3.4. Observed Survival, Expected Survival, and Relative Survival for Patients Who Were Discharged from the Hospital and Were Alive 30 Days after the STEMI

**Figure 4:**
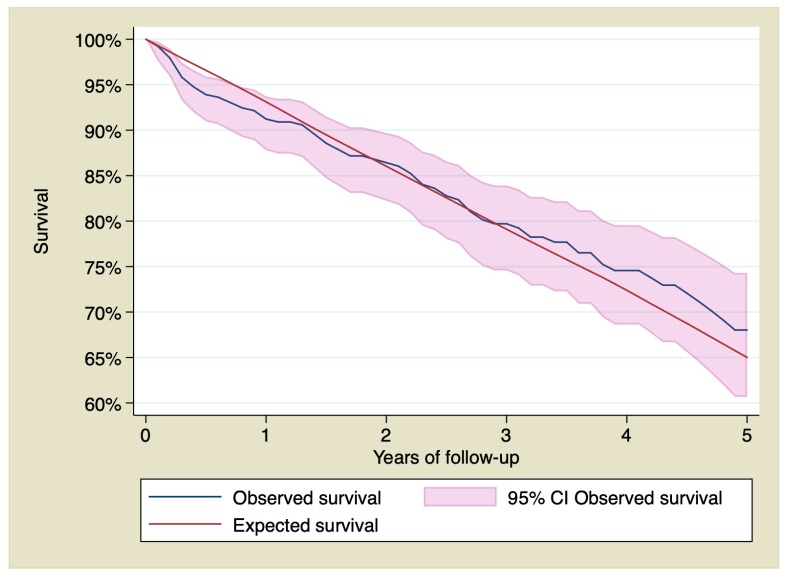
95% confidence interval of the observed survival compared with the expected survival. Only patients who survived the first 30 days after the ST elevation myocardial infarction.

Cumulative survival at 1, 3, and 5 years of follow-up for patients who suffered a STEMI and survived the first 30 days was 91.22% (CI 95% 87.80–93.72), 79.71% (CI 95% 74.58–83.92), and 68.02% (CI 95% 60.66–74.3), whereas in the reference population it is 93.11%, 79.10%, and 65.01%, respectively. Figure 4 shows the survival of the STEMI group compared with the general population for patients who survived the first 30 days.

**Figure 5:**
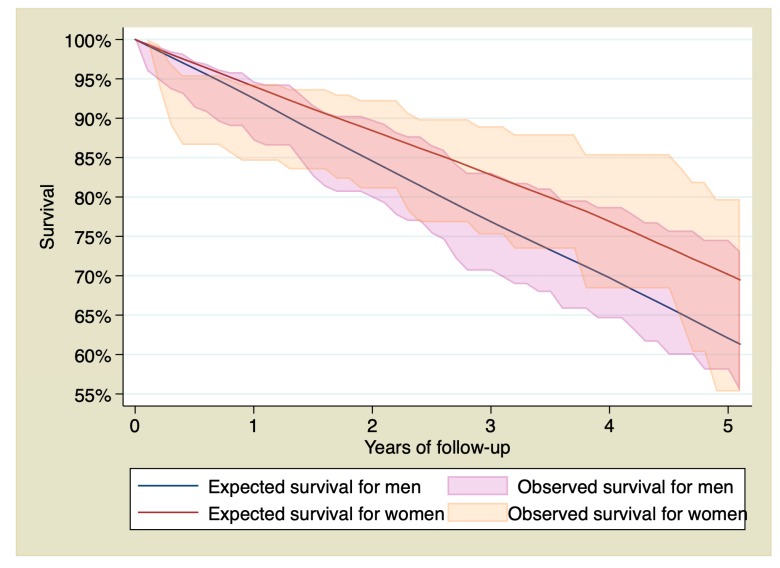
95% confidence interval of the observed survival compared with the expected survival. Only patients who survived the first 30 days after the ST elevation myocardial infarction, stratified by sex.

Stratifying by sex, survival of men with STEMI at 1, 3, and 5 years of follow-up was 91.70% (CI 95% 87.14–94.69), 77.59% (CI 95% 70.64–83.09), and 67.07% (CI 95% 58.06–74.57), whereas in the reference population it was 92.51%, 76.86%, and 62.07%. Survival in women with STEMI was 90.59% (IC 95% 84.59–94.33), 83.33% (IC 95% 75.23–88.98), and 69.34% (IC 95% 55.30–79.75) whereas in the reference population it was 94.05%, 82.81%, and 70.16%. Figure 5 shows the survival curves of patients with STEMI who survived the first 30 days, stratified by sex and compared with the general population.

In men, RS of the first year did not show an excess of mortality due to the STEMI, RS = 98.65% (CI 95% 93.71–101.90%). However, RS of the first year in women did show an excess of mortality with a RS = 95.82% (CI 95% 89.18–99.98). RS of both men and women showed that observed and expected survivals were similar after the first year. Table 5 shows observed and expected cumulative survival and RS stratified by sex and calculated by the year of follow-up.

### 3.5. Influence of Sex on Long-Term Survival

The influence of sex on late survival was assessed by a Cox regression analysis. Sex was not associated with late survival, HR = 1.02 (CI 95% 0.67–1.53; *p* = 0.92). The association of the other possible clinical and angiographic variables with late survival are described in Table 6.

## 4. Discussion

Primary PCI protocols in STEMI have widely demonstrated an improvement in clinical outcomes, including survival [1], particularly when performed in high-volume interventional centers [29,30]. In this context, the main finding of this study is the survival of those patients who were still alive 30 days after the MI. Remarkably, STEMI patients >75 years who underwent PCI in our center and survived the first 30 days, reached long-term survival similar to that of the general population. Our results highlight the importance of primary PCI strategies irrespective of age. Primary PCI in the elderly would let to recover their life expectancy. Interestingly, the first month after PCI seems a critical period in determining the prognosis.

Some studies have shown that female gender is an independent predictor for short-term mortality after primary PCI [31,32]. However, the possible influence of sex on long-term survival and is still unclear. The first month after primary PCI is a high-risk period, for both men and women. This high risk at the beginning makes the observed survival lower than the expected survival for both sexes during the first 3 years of follow-up. However, at a certain time between the third and fourth year of follow-up, the confidence interval of the observed survival curve begins to include the expected survival curve, indicating that both survivals are becoming similar. That means that STEMI patients >75 years old who underwent PCI at our center achieved long-term survival similar to that of the general population with the same age, sex, and geographical location from the third year onwards.

When analyzing differences between men and women from our cohort, we found some remarkable data. Men who survived the first 30 days after the STEMI completely recover their life expectancy, with both observed and expected survival curves similar from the beginning, as if they had never suffered the STEMI. This conclusion could be confirmed with the calculation of the RS. So, RS of the first year in men did not show an excess of mortality due to the STEMI. Conversely, women who survived the first 30 days were still at an increased risk of death during the first year, indicating that the risk of death in this group of patients is not limited to the first 30 days but is longer and more pronounced than that of the men. There were no significant differences in left ventricular ejection fraction that could explain these differences. Our data showed that, after this first year with high risk, women also achieved a survival similar to that of the general population during the rest of the follow-up.

Finally, despite women usually live longer in the general population, our data show that sex is not a risk factor for mortality in this group of patients. This is likely because women have a slightly higher risk of dying than men in the first 30 days of the follow-up. Probably, after having a STEMI, women cease to enjoy a greater long-term survival than men.

### Limitations

The present study has several limitations. Data were collected retrospectively. The results are only applicable to the studied population (STEMI patients > 75 years old who underwent primary PCI in a single academic center). Caution should be taken when extrapolating these results. We did not have data on treatment throughout the follow-up period. In addition, sample size is relatively small and statistical power could be low.

## 5. Conclusions

Life expectancy of both men and women suffering a STEMI and treated with primary PCI is intimately linked to survival during the first period of time. Women who survive the first 30 days after the STEMI still have an excess of mortality due to the STEMI or its consequences during the first year of follow-up. This excess of mortality is not shown in men who survive the first 30 days. After one year, the risk of death for both men and women seems similar to that of the general population of the same age, sex, and geographical region. Sex is not a risk factor for long-term mortality in this group of patients.

## Figures and Tables

**Table 1:** Baseline characteristics.

Variable	Male (*n* = 263)	Female (*n* = 187)	*p*
Age (Years)	81.6 ± 4.7	83.1 ± 4.8	0.001
Hypertension	152 (57.8%)	132 (70.6%)	0.005
Diabetes	84 (31.9%)	52 (27.8%)	0.347
Dyslipidemia	116 (44.1%)	69 (36.8%)	0.126
Smoking Habit	117 (44.5%)	18 (9.6%)	0.001
Chronic Kidney Disease	38 (14.4%)	24 (12.8%)	0.624
Previous Myocardial Infarction	54 (20.5%)	22 (11.8%)	0.014
Previous PCI	39 (14.8%)	18 (9.7%)	0.102
Previous CABG	12 (4.6%)	0 (0%)	0.003

MI: myocardial infarction, PCI: percutaneous coronary intervention, CABG: coronary artery bypass grafting.

**Table 2:** Intra-procedural characteristics.

Variable	Male (*n* = 263)	Female (*n* = 187)	*p*
Access:			0.894
Femoral	113 (42.9%)	81 (42.8%)	
Radial	148 (56.3%)	106 (56.7%)	
Braquial	2 (0.8%)	0 (0%)	
Culprit Artery:			0.147
Left Main	13 (4.9%)	3 (1.6%)	
LAD	132 (50.2%)	86 (46%)	
LCX	39 (14.8%)	37 (19.8%)	
RCA	75 (28.5%)	61 (32.6%)	
Graft	4 (1.5%)	0 (0%)	
Multivessel Disease	141 (53.6%)	72 (38.5%)	0.002
Stents Implanted	1.3 ± 0.8	1.3 ± 0.9	0.501
IABP	14 (5.3%)	11 (5.9%)	0.799
Failed PCI	11 (4.2%)	8 (4.3%)	0.960
Killip Kimball Class			0.684
I	182 (69.2%)	126 (67.4%)	
II	39 (14.8%)	31 (16.6%)	
III	16 (6.1%)	8 (4.3%)	
IV	26 (9.9%)	22 (11.8%)	
Vascular Complications	4 (1.5%)	5 (2.7%)	0.389
Arrhythmia	15 (5.7%)	10 (5.3%)	0.871
Endotracheal Intubation	4 (1.5%)	7 (3.7%)	0.132
US TnT (pg/mL)	6175 ± 547	5112 ± 507	0.169
LVEF at Discharge	48.93 ± 11.3	49.56 ± 11.5	0.584
CI-AKI	61 (23.2%)	48 (25.7%)	0.546
Procedural Death	3 (1.1.%)	4 (2.1%)	0.399

LAD: left anterior descending coronary artery. LCX: left circumflex coronary artery. RCA: right coronary artery. IABP: intra-aortic balloon pump. PCI: percutaneous coronary intervention. US TnT: ultra-sensitive troponin T. LVEF: left ventricle ejection fraction. CI-AKI: contrast-induced acute kidney injury.

**Table 3:** Causes of death.

Variable		
**Deaths < 30 days (*n* = 59)**	**Male (*n* = 31)**	**Female (*n* = 28)**
Cardiovascular	25 (80.7%)	25 (89.3%)
Stroke	0 (0%)	1 (3.6%)
Major Bleeding	0 (0%)	0 (0%)
Infection	1(3.2%)	0 (0%)
Others	5 (16.1%)	2 (7.1%)
**Deaths after Discharge or >30 days (*n* = 82)**	**Male (*n* = 55)**	**Female (*n* = 27)**
Cardiovascular	16 (29.1%)	11 (40.7%)
Stroke	4 (7.2%)	4 (14.8%)
Major Bleeding	0 (0%)	1 (3.7%)
Sepsis	6 (10.9%)	2 (7.4%)
Respiratory Infection	6 (10.9%)	2 (7.4%)
Cancer	5 (9.1%)	2 (7.4%)
Unknown	5 (9.1%)	1 (3.7%)
Others	13 (23.6%)	4 (14.8%)

**Table 4:** Observed and expected survival during the follow-up stratified by sex and calculated by year of follow-up. Relative survival by annual intervals were also calculated. CI: Confidence Interval; STEMI: ST elevation myocardial infarction.

Year of Follow-Up	Cumulative Survival of Patients with STEMI (Observed Survival)	Cumulative Survival in the Reference Group (Expected Survival)	Annual Relative Survival *
Men			
First Year	82.39% (CI 95% 77.05–86.59)	92.51%	88.06% (CI 95% 82.15–92.73)
Second Year	76.93% (CI 95% 70.96–81.83)	84.55%	101.77% (CI 95% 96.28–104.94)
Third Year	69.71% (CI 95% 62.95–75.48%)	76.86%	99.27% (CI 95% 92.16–103.55)
Fourth Year	65.03% (CI 95% 57.67–71.43)	69.74%	102.37% (CI 95% 93.92–106.4)
Fifth Year	60.26% (CI 95% 51.97–67.58%)	62.06%	102.96% (CI 95% 89.99–108.3)
Women			
First Year	80.44% (CI 95% 73.60–85.62)	94.06%	83.89% (CI 95% 76.31–89.75)
Second Year	78.01% (CI 95% 70.85–83.62)	88.43%	103.06% (CI 95% 96.73–105.19)
Third Year	74% (CI 95% 66.04–80.37)	82.81%	100.99% (CI 95% 92.67–104.3)
Fourth Year	69.54% (CI 95% 60.34–77.01)	77.56%	101.02% (CI 95% 89.27–105.13)
Fifth Year	61.57% (CI 95% 49.24–71.74)	70.27%	97.41% (CI 95% 77.10–105.03)

* Relative survival calculated by interval. This is not a cumulative estimation.

**Table 5:** Observed and expected survival during the follow-up stratified by sex and calculated by year of follow-up for patients who survived the first 30 days after the STEMI. Relative survival by annual intervals are also calculated.

Year of Follow-Up	Cumulative Survival of Patients with STEMI (Observed Survival)	Cumulative Survival in the Reference Population (Expected Survival)	Annual Relative Survival *
Men			
First Year	91.70% (CI 95% 87.14–94.69)	92.51%	98.65% (CI 95% 93.71–101.90)
Second Year	85.63% (CI 95% 79.92–89.81)	84.56%	101.77% (CI 95% 96.28–104.94)
Third Year	77.59% (CI 95% 70.64–83.09)	76.86%	99.27% (CI 95% 92.16–103.55)
Fourth Year	72.38% (CI 95% 64.59–78.74)	69.74%	102.37% (CI 95% 93.92–106.4)
Fifth Year	67.07% (CI 95% 58.06–74.57)	62.07%	102.96% (CI 95% 89.99–108.3)
Women			
First Year	90.59% (CI 95% 84.59–94.33)	94.05%,	95.82% (CI 95% 89.18–99.98)
Second Year	87.85% (CI 95% 81.07–92.32)	88.42%	103.06% (CI 95% 96.73–105.19)
Third Year	83.33% (CI 95% 75.23–88.98)	82.81%	100.99% (CI 95% 92.67–104.3)
Fourth Year	78.31% (CI 95% 68.38–85.45)	76.89%	101.02% (CI 95% 89.27–105.13)
Fifth Year	69.34% (CI 95% 55.30–79.75)	70.16%	97.41% (CI 95% 77.10–105.03)

* Relative survival calculated by interval. This is not a cumulative estimation.

**Table 6:** Multivariable analysis.

Variable	HR	95% Confidence Interval	*p*
Sex	1.02	0.67–1.53	0.920
Age	1.06	1.02–1.1	0.004
Diabetes	0.93	0.63–1.38	0.720
Hypertension	1.19	0.80–1.78	0.390
Dyslipidemia	0.74	0.49–1.1	0.140
Smoking History	1.23	0.81–1.88	0.340
Chronic Kidney Disease	1.54	1.01–2.30	0.046
Previous Myocardial Infarction	0.66	0.32–1.35	0.250
Previous Coronary Angioplasty	1.19	0.53–2.71	0.660
Previous Coronary Artery Bypass Surgery	2.34	0.98–5.74	0.054
Lesion on Anterior Descending Artery	0.95	0.66–1.39	0.800
III or IV Killip Class	5.14	3.41–7.75	<0.001
Multivessel Diseas	1.09	0.76–1.58	0.630

HR: Hazard Ratio.
